# Supplementary material for: Bacterial chromatin remodeling associated with transcription-induced domains at pathogenicity Islands
Source: Nat Commun. 2026 Jan 8;17:161. doi: 10.1038/s41467-025-67746-w (PMC12783615; doi:10.1038/s41467-025-67746-w)
Supplement: Supplementary file 1 — Supplementary Information [file 41467_2025_67746_MOESM1_ESM.pdf]

Supplementary Information

Bacterial chromatin remodeling associated with transcription-induced domains at pathogenicity Islands.

Mounia Kortebi[1], Mickael Bourge[1], Romain Le Bars[1], Erwin Van Dijk[1], Charles J. Dorman[2], Stéphanie Bury-Moné[1], Frédéric Boccard[1] and Virginia S. Lioy[1*]

[1]Université Paris-Saclay, CEA, CNRS, Institute for Integrative Biology of the Cell (I2BC), 91198, Gif-sur-Yvette, France

[2] Department of Microbiology, Moyne Institute of Preventive Medicine, Trinity College Dublin, Dublin 2, Ireland

*Correspondence: virginia.lioy@i2bc.paris-saclay.fr

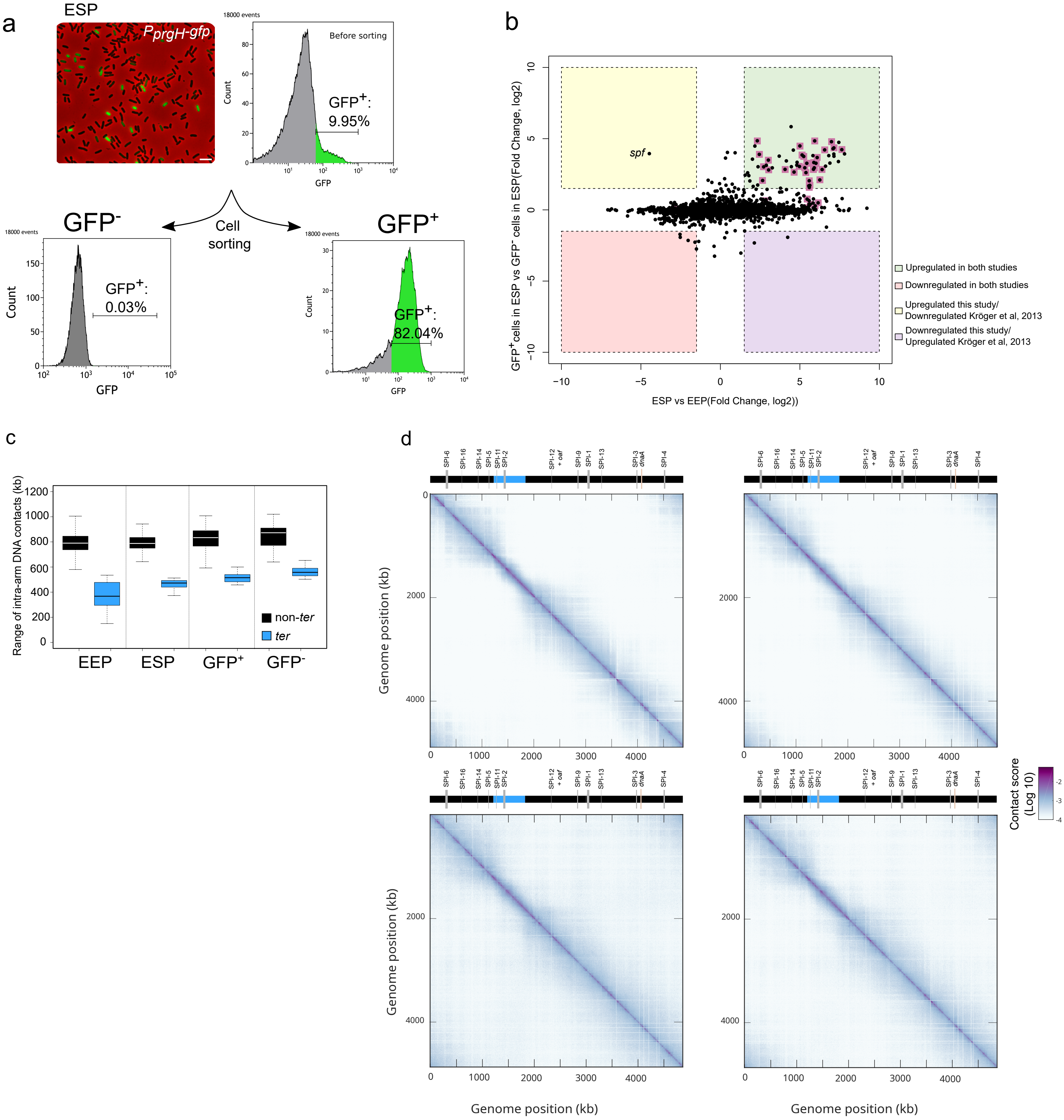
a
ESP
PprgH-gfp
18000 events
Before sorting
Count
GFP
GFP+:
9.95%
Cell
sorting
GFP-
GFP+
GFP+:
0.03%
GFP+:
82.04%
b
GFP+cells in ESP vs GFP- cells in ESP(Fold Change, log2)
ESP vs EEP(Fold Change, log2))
spf
Upregulated in both studies
Downregulated in both studies
Upregulated this study/
Downregulated Kröger et al, 2013
Downregulated this study/
Upregulated Kröger et al, 2013
c
Range of intra-arm DNA contacts (kb)
non-ter
ter
EEP
ESP
GFP+
GFP-
d
SPI-6
SPI-16
SPI-14
SPI-5
SPI-11
SPI-2
SPI-12
+ oaf
SPI-9
SPI-1
SPI-13
SPI-3
dnaA
SPI-4
Genome position (kb)
Contact score
(Log 10)

Supplementary Figure 1. Transcriptomic analyses and chromosome folding in *Salmonella* cells.

a. Bistable expression of P_{prgH}-*gfp* in *S*. Typhimurium as determined by fluorescence microscopy (upper left) or FACS (upper right). Below, expression of P_{prgH}-*gfp* after cell sorting in the GFP negative (GFP^-) and GFP positive (GFP^+) populations.
b. Comparative analysis of genes that are significantly upregulated in this study (GFP^+ *vs* GFP^-) and in a previous study[1]. SPI-1 genes are highlighted in pink.
c. Quantification of the range of intra-arm DNA contacts within *ter* or non-*ter* regions for *S*. Typhimurium Hi-C contact maps. Quantification was performed as previously done in[2]. All boxplots of this figure represent the first quartile, median and third quartile. The upper whisker extends from the hinge to the largest value no further than 1.5* the inter-quartile range (IQR, i.e. distance between the first and third quartiles) from the hinge. The lower whisker extends from the hinge to the smallest value at most 1.5*IQR of the hinge. For clarity, outliers were excluded from these graphical representations (but were considered for the numerical exploitation of the data). Source data are provided as a Source Data file.
d. Normalized contact maps obtained in asynchronous populations of *Salmonella* in early exponentially phase (EEP, upper-left panel), early stationary phase (ESP, upper-right panel), anaerobic growth (lower-left panel) and under oxygen shock (lower right panel).

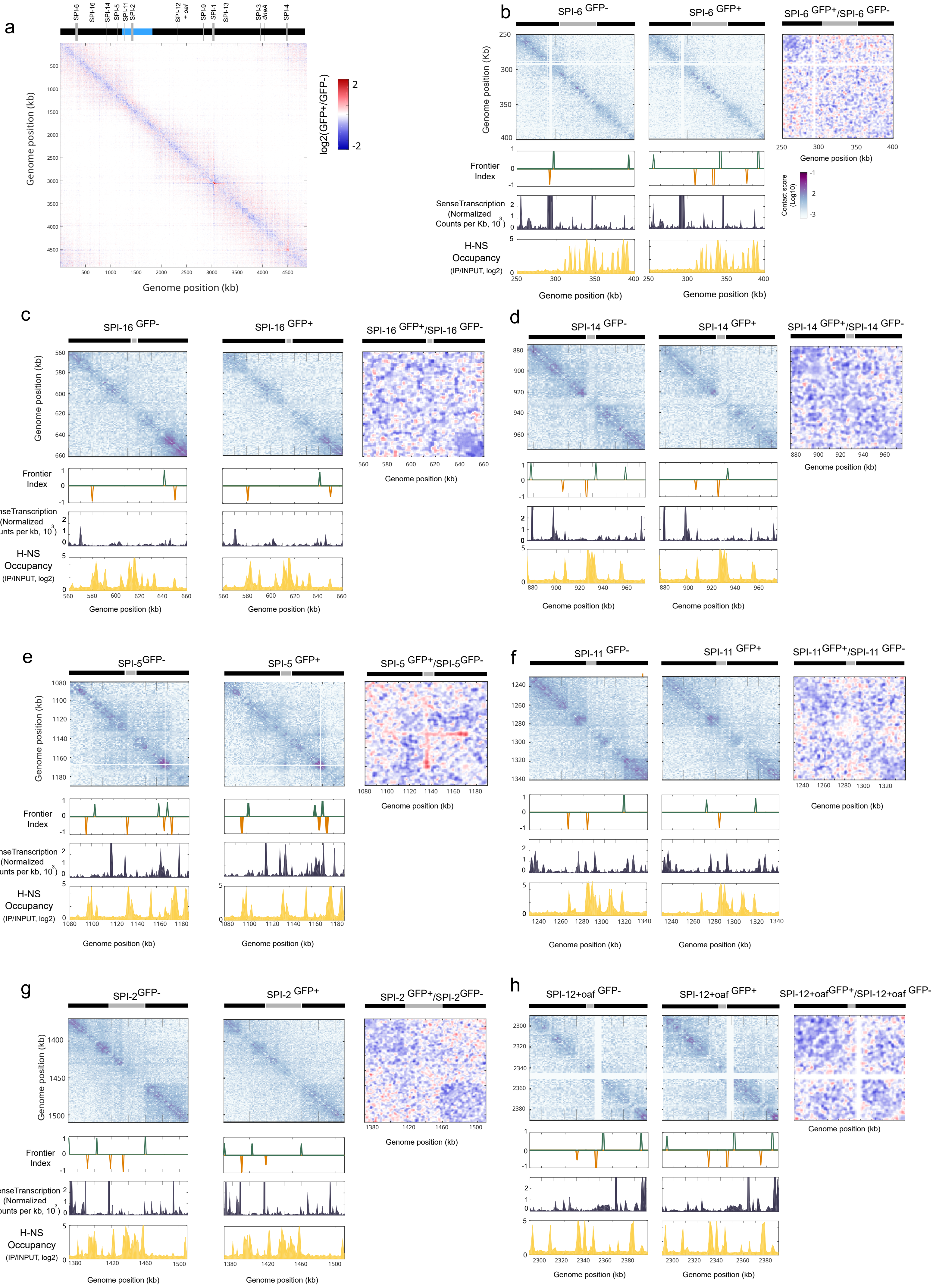

a
SPI-6
SPI-16
SPI-14
SPI-5
SPI-11
SPI-2
SPI-12 + oaf
SPI-9
SPI-1
SPI-13
SPI-3 dnaA
SPI-4
Genome position (kb)
log2(GFP+/GFP-)
b
SPI-6 GFP-
SPI-6 GFP+
SPI-6 GFP+/SPI-6 GFP-
Frontier Index
SenseTranscription (Normalized Counts per Kb, 10³)
H-NS Occupancy (IP/INPUT, log2)
Contact score (Log10)
c
SPI-16 GFP-
SPI-16 GFP+
SPI-16 GFP+/SPI-16 GFP-
d
SPI-14 GFP-
SPI-14 GFP+
SPI-14 GFP+/SPI-14 GFP-
e
SPI-5GFP-
SPI-5 GFP+
SPI-5 GFP+/SPI-5GFP-
f
SPI-11 GFP-
SPI-11 GFP+
SPI-11GFP+/SPI-11 GFP-
g
SPI-2GFP-
SPI-2 GFP+
SPI-2 GFP+/SPI-2GFP-
h
SPI-12+oaf GFP-
SPI-12+oaf GFP+
SPI-12+oafGFP+/SPI-12+oaf GFP-

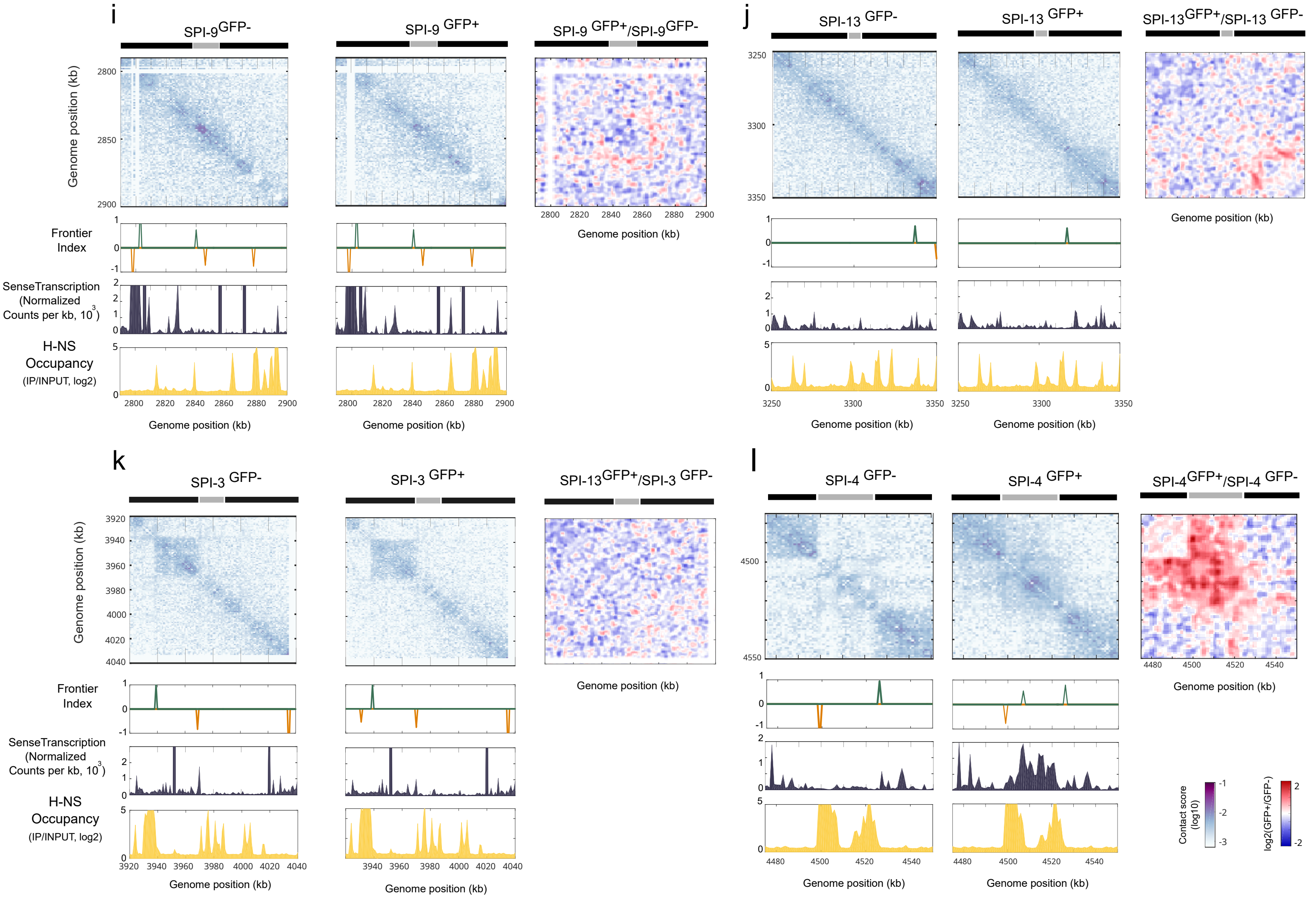

i
SPI-9 GFP-
SPI-9 GFP+
SPI-9 GFP+/SPI-9 GFP-
Genome position (kb)
Frontier Index
SenseTranscription (Normalized Counts per kb, 10^3)
H-NS Occupancy (IP/INPUT, log2)
j
SPI-13 GFP-
SPI-13 GFP+
SPI-13 GFP+/SPI-13 GFP-
k
SPI-3 GFP-
SPI-3 GFP+
SPI-13 GFP+/SPI-3 GFP-
l
SPI-4 GFP-
SPI-4 GFP+
SPI-4 GFP+/SPI-4 GFP-
Contact score (log10)
log2(GFP+/GFP-)

Supplementary Figure 2: Chromatin organization and gene expression in other SPIs.

a. Ratio of normalized contact maps for GFP^+ and GFP^- populations (log2). A decrease or increase in contacts in the GFP^+ population compared with the GFP^- population is represented with a blue or red color, respectively. White indicates no differences between the two populations.
a. to (l) Contact maps, Frontier Index, gene expression and H-NS occupancy in the GFP^- (left panel), GFP^+ (right panel), and normalized contact ratios centered in the indicated SPIs. Please note that the P_{prgH}-*gfp* reporter cassette used to sort GFP^+ cells is located downstream SPI-5, in between the region in which SPI-5 forms a loop. Source data are provided as a Source Data file.

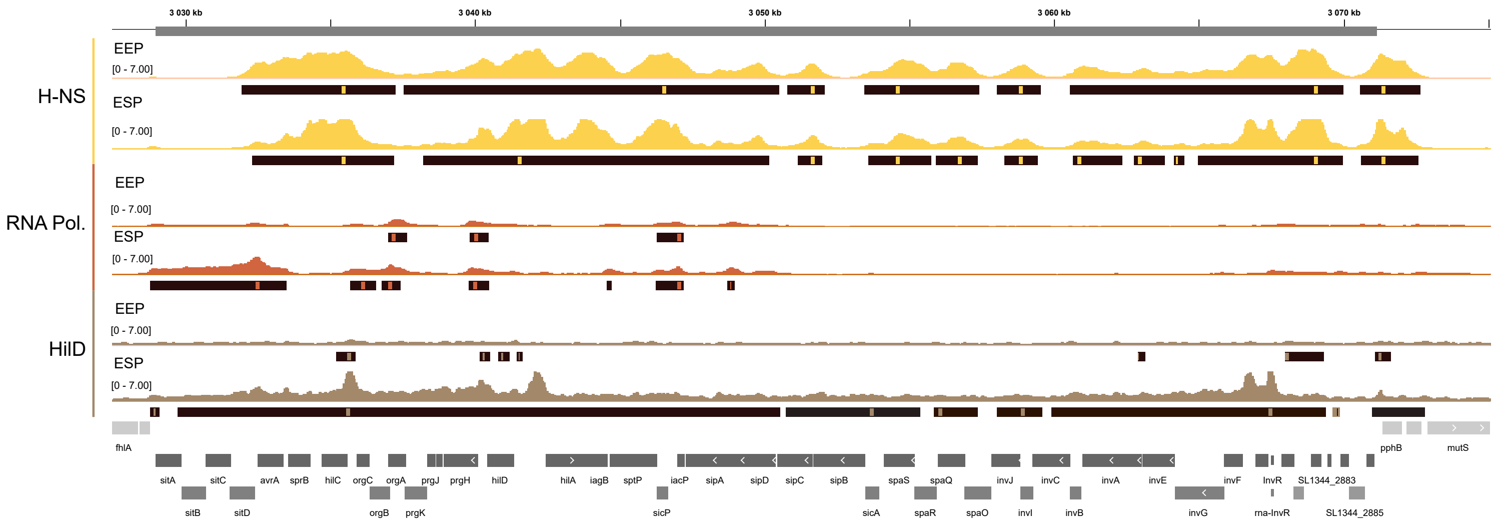

3 030 kb
3 040 kb
3 050 kb
3 060 kb
3 070 kb
H-NS
RNA Pol.
HilD
EEP
ESP
[0 - 7.00]
fhlA
sitA
sitB
sitC
sitD
avrA
sprB
hilC
orgC
orgB
orgA
prgJ
prgK
prgH
hilD
hilA
iagB
sptP
iacP
sicP
sipA
sipD
sipC
sipB
sicA
spaS
spaR
spaQ
spaO
invJ
invI
invC
invB
invA
invE
invG
invF
InvR
rna-InvR
SL1344_2883
SL1344_2885
pphB
mutS

Supplementary Figure 3: Protein occupancy within SPI-1 in non-sorted *Salmonella* populations.

IGV snapshots centered on SPI-1 showing the binding occupancy of H-NS, RNA Polymerase (RNA Pol.) and HilD in repressed (EEP) or active SPI-1 (ESP) conditions. The histograms show the log2 (ChIP/Input) for each binding factor. Below the histograms, significant peaks detected by MACS2 are highlighted in black. Bottom plot shows SPI-1 genes and their neighboring genetic context.

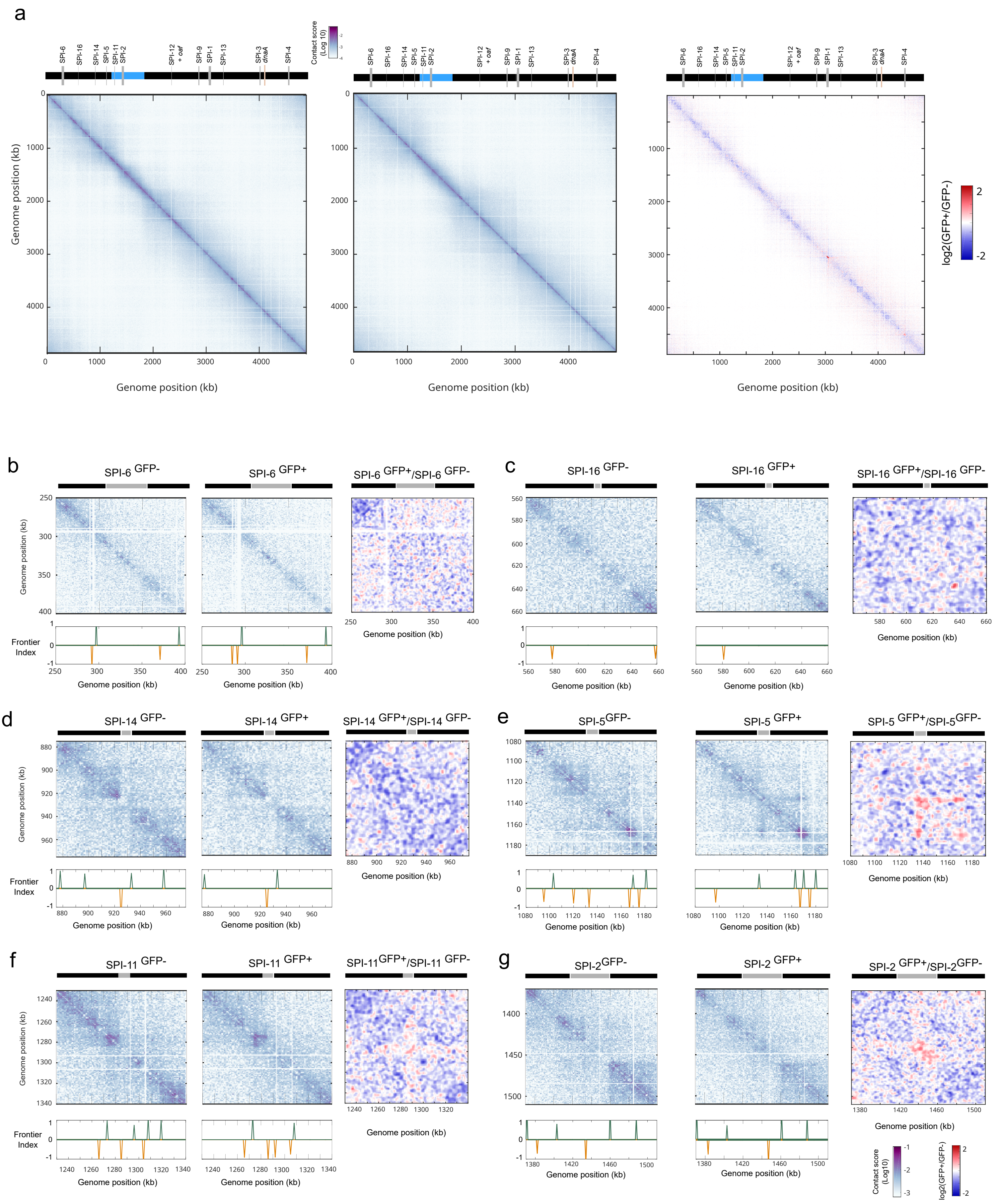

a
SPI-6
SPI-16
SPI-14
SPI-5
SPI-11
SPI-2
SPI-12 + oaf
SPI-9
SPI-1
SPI-13
SPI-3 dnaA
SPI-4
Contact score (Log 10)
Genome position (kb)
log2(GFP+/GFP-)
b
SPI-6 GFP-
SPI-6 GFP+
SPI-6 GFP+/SPI-6 GFP-
Frontier Index
c
SPI-16 GFP-
SPI-16 GFP+
SPI-16 GFP+/SPI-16 GFP-
d
SPI-14 GFP-
SPI-14 GFP+
SPI-14 GFP+/SPI-14 GFP-
e
SPI-5GFP-
SPI-5 GFP+
SPI-5 GFP+/SPI-5GFP-
f
SPI-11 GFP-
SPI-11 GFP+
SPI-11GFP+/SPI-11 GFP-
g
SPI-2GFP-
SPI-2 GFP+
SPI-2 GFP+/SPI-2GFP-
log2(GFP+/GFP-)

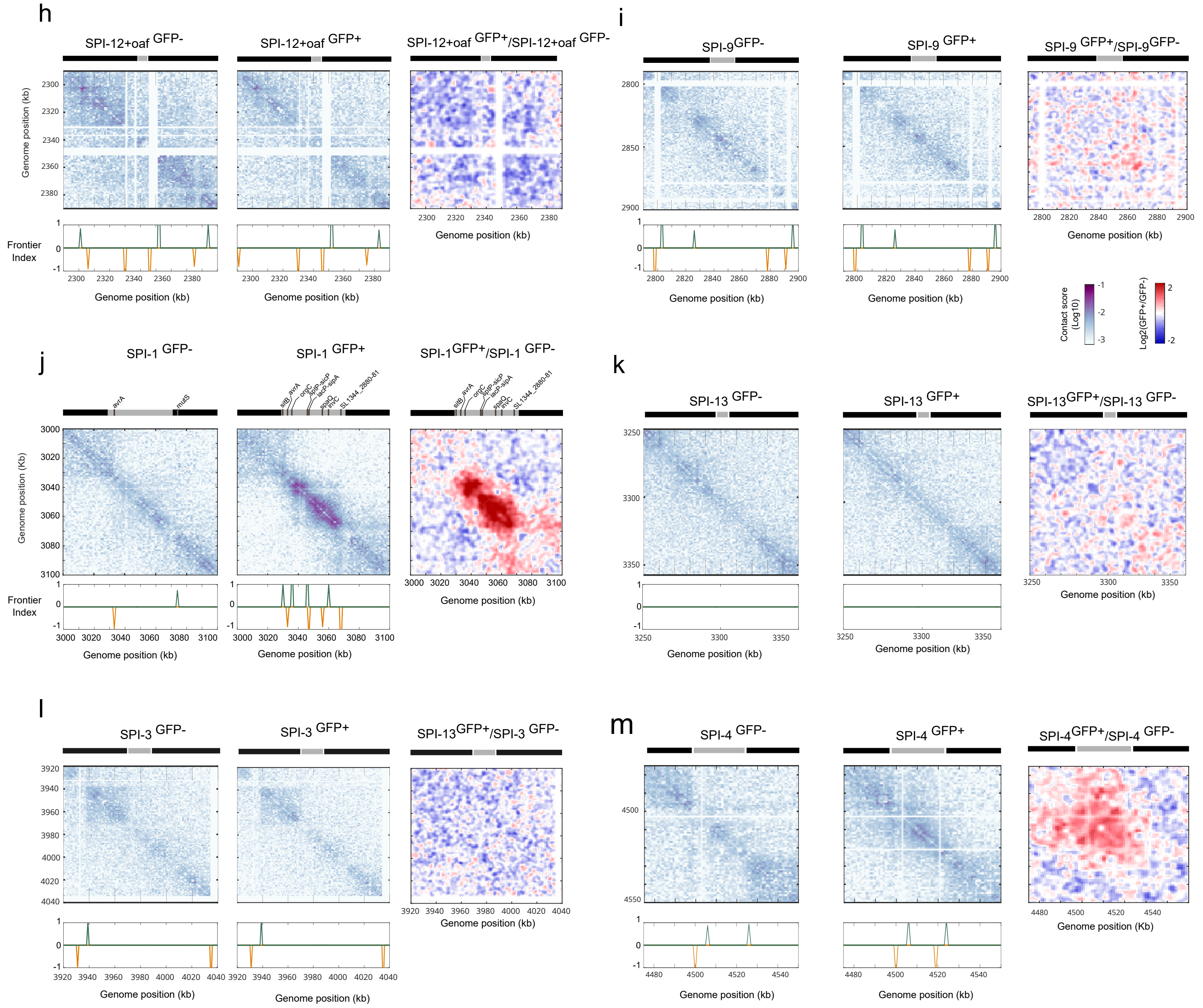
h
SPI-12+oaf GFP-
SPI-12+oaf GFP+
SPI-12+oaf GFP+/SPI-12+oaf GFP-
Genome position (kb)
Frontier Index
i
SPI-9GFP-
SPI-9 GFP+
SPI-9 GFP+/SPI-9GFP-
Contact score (Log10)
Log2(GFP+/GFP-)
j
SPI-1 GFP-
SPI-1 GFP+
SPI-1GFP+/SPI-1 GFP-
avrA
mutS
sitB
orgC
sptP-sicP
iacP-sipA
spaQ
invC
SL1344_2880-81
Genome position (Kb)
k
SPI-13 GFP-
SPI-13 GFP+
SPI-13GFP+/SPI-13 GFP-
l
SPI-3 GFP-
SPI-3 GFP+
SPI-13GFP+/SPI-3 GFP-
m
SPI-4 GFP-
SPI-4 GFP+
SPI-4GFP+/SPI-4 GFP-
Genome position (Kb)

Supplementary Figure 4. **Chromosome organization in sorted GFP- and GFP+ populations in the absence of HilE**

a. 3D *Salmonella* chromosome folding in *hilE* deletion mutant cells in GFP- (Left panel) or GFP+ (middle panel) populations. Ratio of normalized contact maps for GFP+ and GFP- populations (Log2, right panel). A decrease or increase in contacts in the GFP+ population compared with the GFP-population is represented with a blue or red color, respectively. White indicates no differences between the two populations.
b. to (m) Contact map, Frontier Index in the *hilE* GFP^- (left panel), *hilE* GFP^+ population (middle panel), and normalized contact ratios (right panel) centered in all the SPIs. Please note that the P_{prgH}-*gfp* reporter cassette used to sort GFP^+ cells is located downstream SPI-5, in between the region in which SPI-5 forms a loop. Source data are provided as a Source Data file

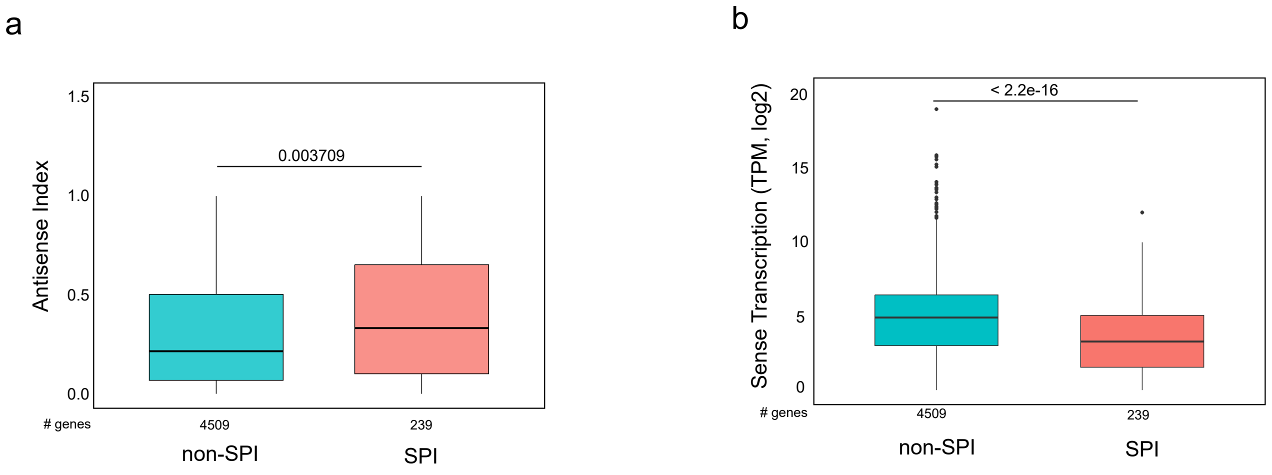

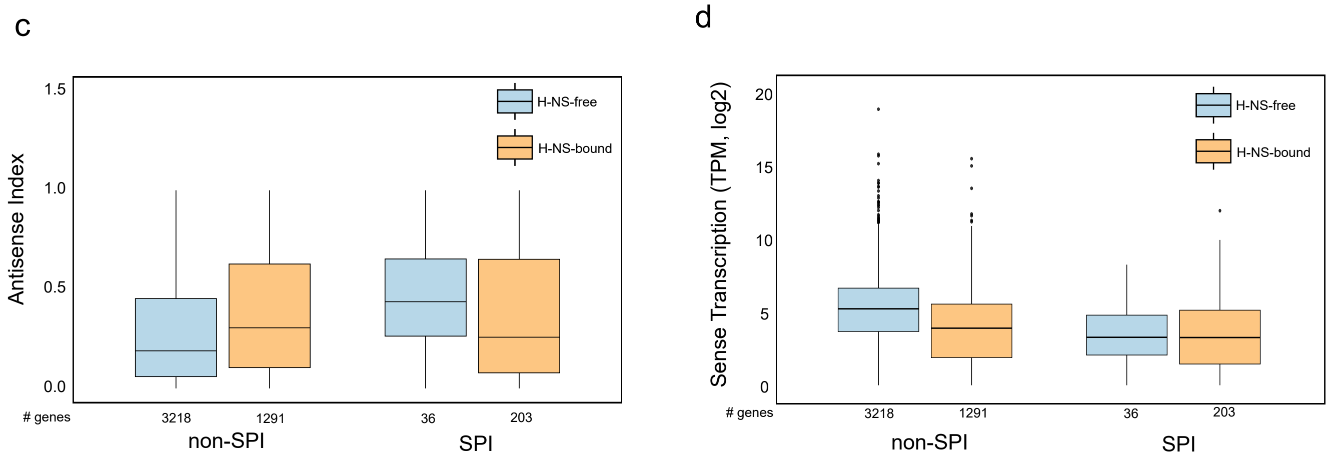

p-values

	non-SPI H-NS-free	non-SPI H-NS-bound	SPI H-NS-free
non-SPI H-NS-bound	**< 2e-16**	NA	NA
SPI H-NS-free	**0.00022**	**0.04602**	NA
SPI H-NS-bound	**0.01362**	0.38909	**0.04602**

p-values

	non-SPI HNS-free	non-SPI HNS-positive	SPI HNS-free
non-SPI HNS-positive	**< 2e-16**	NA	NA
SPI HNS-free	**8.3e-05**	0.4654	NA
SPI HNS-positive	**< 2e-16**	**0.0061**	0.6703

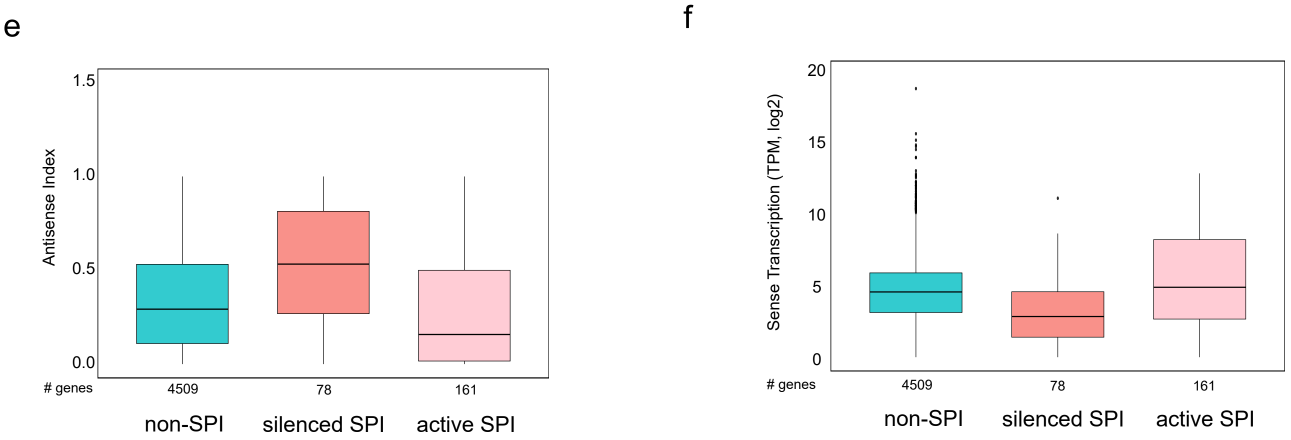

p-values

	non-SPI	silenced SPI
silenced SPI	**4.7e-08**	NA
active SPI	**2.5e-06**	**4.6e-09**

p-values

	non-SPI	silenced SPI
silenced SPI	**5.2e-09**	NA
active SPI	0.053	**3.6e-06**

Supplementary Figure 5. Level of transcription in non-SPI and SPI genes in GFP$^-$ and GFP$^+$ cells in ESP.

a. Antisense transcriptional activity within non-SPIs (turquoise) and SPI (red) genes in GFP$^-$ cells in ESP.
b. Sense transcriptional activity within non-SPIs (turquoise) and SPI (red) genes in GFP$^-$ cells in ESP.
c. Antisense transcriptional activity within non-SPI genes and SPI genes either free of H-NS (light blue) or bound by H-NS (yellow) in GFP$^-$ cells grown in ESP.
d. Sense transcriptional activity within non-SPI genes and SPI genes either free of H-NS (light blue) or bound by H-NS (yellow) in GFP$^-$ cells grown in ESP.
e. Antisense transcriptional activity within non-SPIs (turquoise) silent SPIs (red) and active SPIs (pink) in GFP$^+$ cells in ESP.
f. Sense transcriptional activity within non-SPIs (turquoise) silent SPIs (red) and active SPIs (pink) in GFP$^+$ cells in ESP.

In all the panels, pairwise comparisons were performed using the two-sided Wilcoxon rank sum test with continuity correction to assess the statistical significance (p<0.05). The number of analysed genes (‘#’) per feature is also indicated. All boxplots of this figure represent the first quartile, median and third quartile. The upper whisker extends from the hinge to the largest value no further than 1.5* the inter-quartile range (IQR, i.e. distance between the first and third quartiles) from the hinge. The lower whisker extends from the hinge to the smallest value at most 1.5*IQR of the hinge.

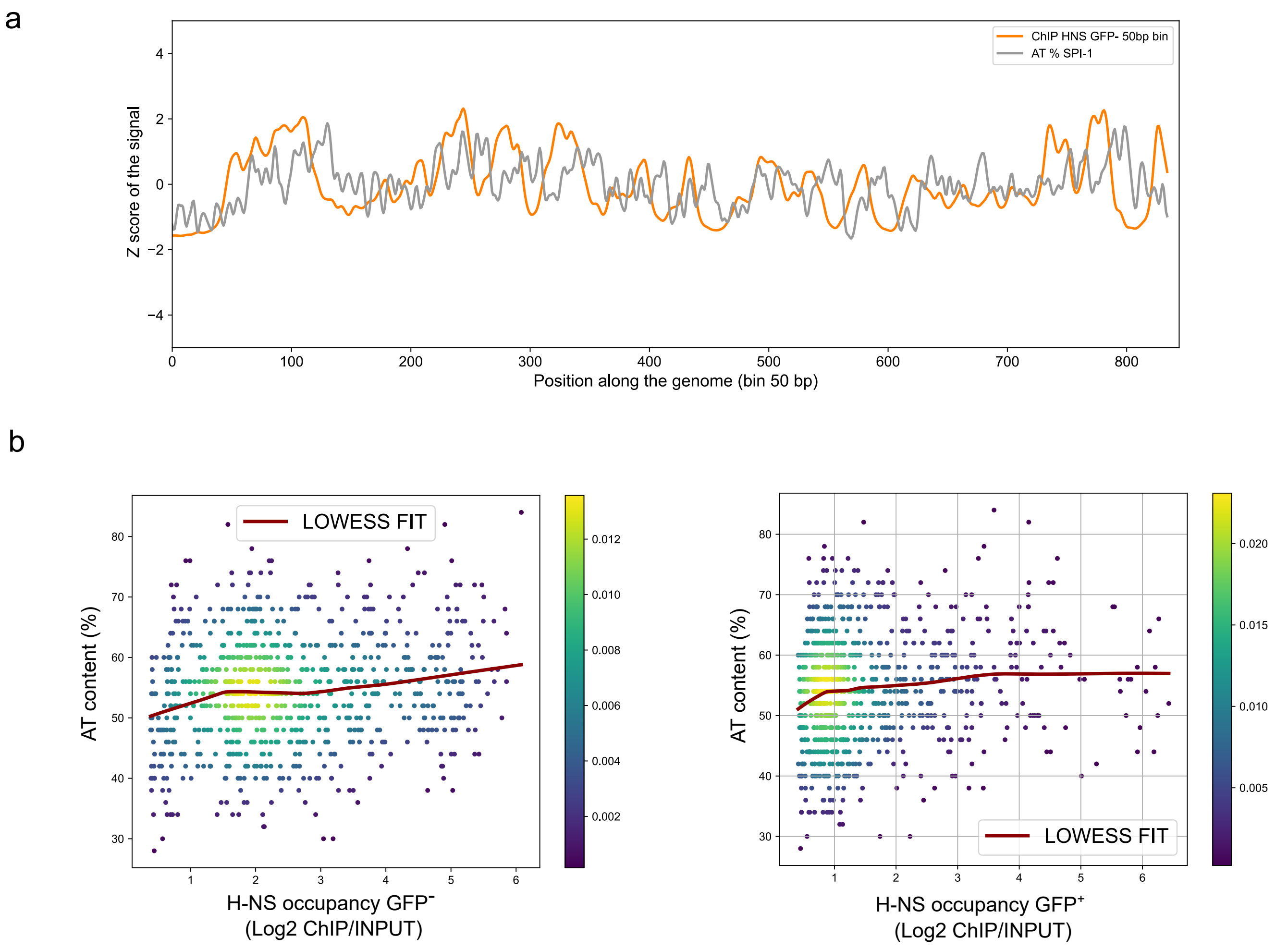

a
ChIP HNS GFP- 50bp bin
AT % SPI-1
Z score of the signal
Position along the genome (bin 50 bp)
b
LOWESS FIT
AT content (%)
H-NS occupancy GFP⁻
(Log2 ChIP/INPUT)
LOWESS FIT
AT content (%)
H-NS occupancy GFP⁺
(Log2 ChIP/INPUT)

Supplementary Figure 6: Correlation between H-NS occupancy and percentage of AT content within SPI-1 in sorted populations of *Salmonella*.

a. Correlation between H-NS occupancy in the GFP^- population (log2(ChIP/Input)) and AT content (%) within SPI-1. AT content was computed using a sliding window of 50 bp in size with an increasing step of 50bp.
b. H-NS abundance as a function of AT content in GFP^- (left panel) or GFP^+ (Right panel) populations.

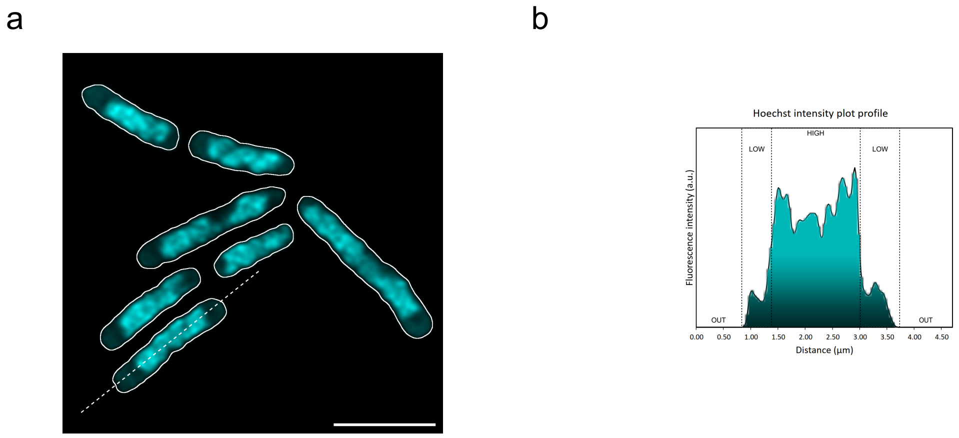
a
b
Hoechst intensity plot profile
OUT
LOW
HIGH
LOW
OUT
Fluorescence intensity (a.u.)
0.00 0.50 1.00 1.50 2.00 2.50 3.00 3.50 4.00 4.50
Distance (μm)

Supplementary Figure 7. 3D SIM Imaging of *Salmonella*.

a Single plane SIM^2 image of Hoechst labelled bacterial DNA (scale bar = 2µm).
b Related intensity plot profile, corresponding to the dotted line drawn on the image in (a).

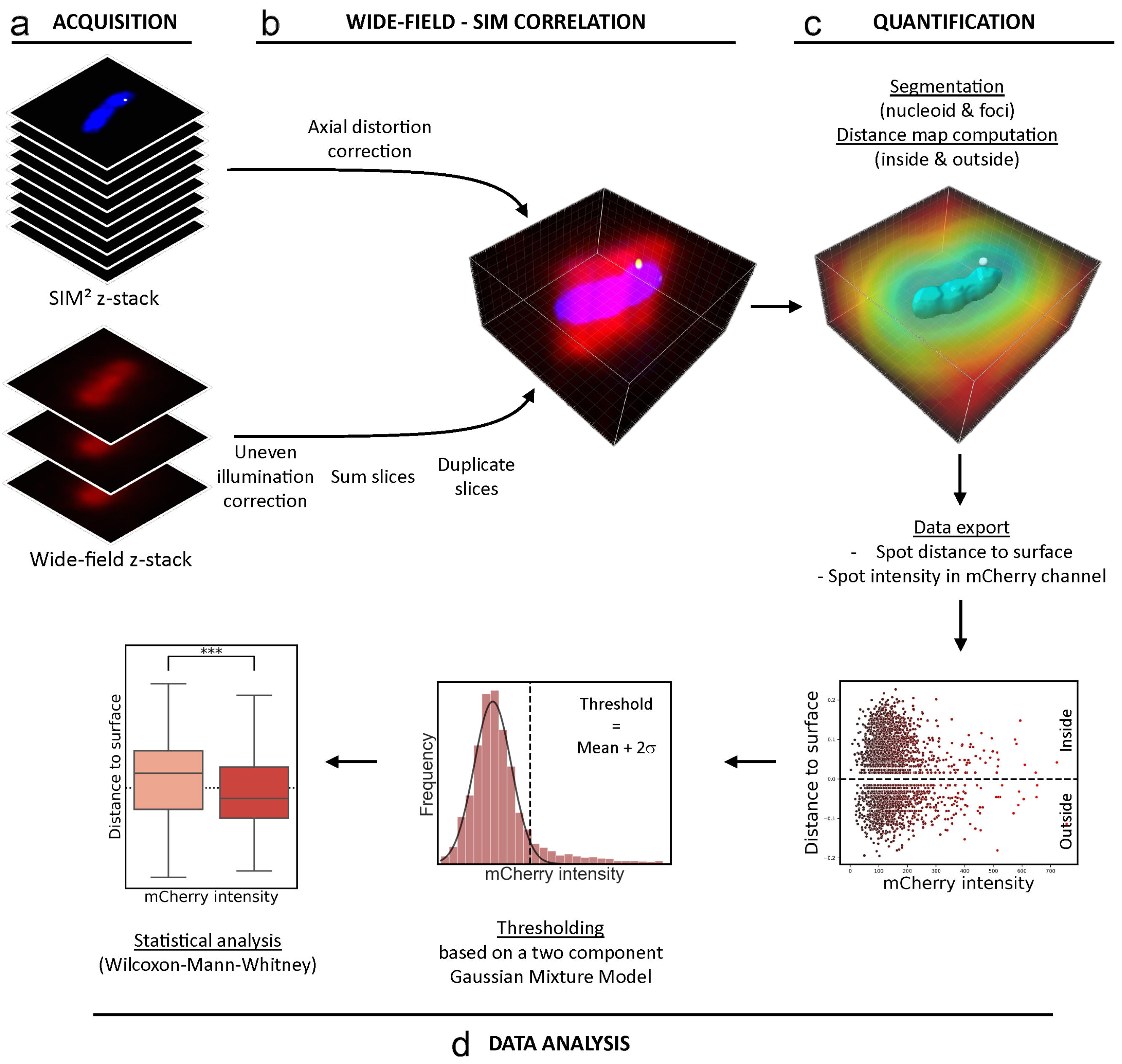
a ACQUISITION
b WIDE-FIELD - SIM CORRELATION
c QUANTIFICATION
SIM² z-stack
Wide-field z-stack
Axial distortion correction
Uneven illumination correction
Sum slices
Duplicate slices
Segmentation (nucleoid & foci)
Distance map computation (inside & outside)
Data export
- Spot distance to surface
- Spot intensity in mCherry channel
Distance to surface
Inside
Outside
mCherry intensity
Frequency
Threshold = Mean + 2σ
***
Statistical analysis (Wilcoxon-Mann-Whitney)
Thresholding based on a two component Gaussian Mixture Model
d DATA ANALYSIS

Supplementary Figure 8. 3D SIM data analysis.

a Cells were acquired in Z stacks that included the whole bacteria, by switching between SIM^2 Z-stacks and wide-field Z-stacks.

b Wide-Field & SIM correlation process: Following the acquisition steps, the wide-field and SIM^2 images were subjected to different processing procedures. The Hoechst and YFP SIM^2 image stacks were corrected for Z distortion. Z-axis intensity profiles were produced transversely to the bacteria (approximately 10 per field) in order to estimate the Z distortion after thresholding and fitting the resulting signals by an ellipse. This factor was used to correct the Z step and thus obtain isotropic cross-sections of the bacteria, limiting errors in 3D distance calculations. The mCherry wide-field stack, employed subsequently for the purpose of intensity quantification, was corrected for uneven illumination: a Rhodamine B solution was imaged under identical conditions to serve as a reference. After background subtraction, the images were divided by this reference image. To avoid errors in correlating the wide field data with the SIM data, a z-projection of the wide-field stack was performed and later duplicated multiple times in order to match the number of planes in the SIM image.

c Quantification: Following the merging of the data, the data set was processed for segmentation. The Hoechst channel was treated to generate surfaces representing all regions of high DNA density, while the YFP channel was used to identify ParB:YFP/*parS*^{pMT1} foci as spots. To remove aberrant foci detections, we kept them for the analysis only if they were closer than 0.2 µm from a nucleoid surface (representing more than 98% of the total detected foci). Two distance maps were then generated based on the surfaces previously generated, with measurements taken towards the inside and outside. The distance between a focus and the closest surface was obtained by measuring the value of each spot in the distance map channel. To determine whether a focus belonged to a SPI-1 ON or SPI-1 OFF cell, the value of the spot in the mCherry projection channel was measured.

d Data Analysis: In order to identify the threshold between the SPI-1ON and SPI-1 OFF populations, a Gaussian Mixture Model based on two components (Python, Scikit-learn library) was employed on a histogram presenting spot intensities in the mCherry channel. The Gaussian of the lowest population (comprising 90% of the spots) was used to determine the threshold with precision by calculating the mean of the Gaussian fit plus twice its standard deviation. Following the splitting of the data into two populations, the spot distances from the DNA-dense regions were subjected to a statistical analysis using the Wilcoxon-Mann-Whitney test.

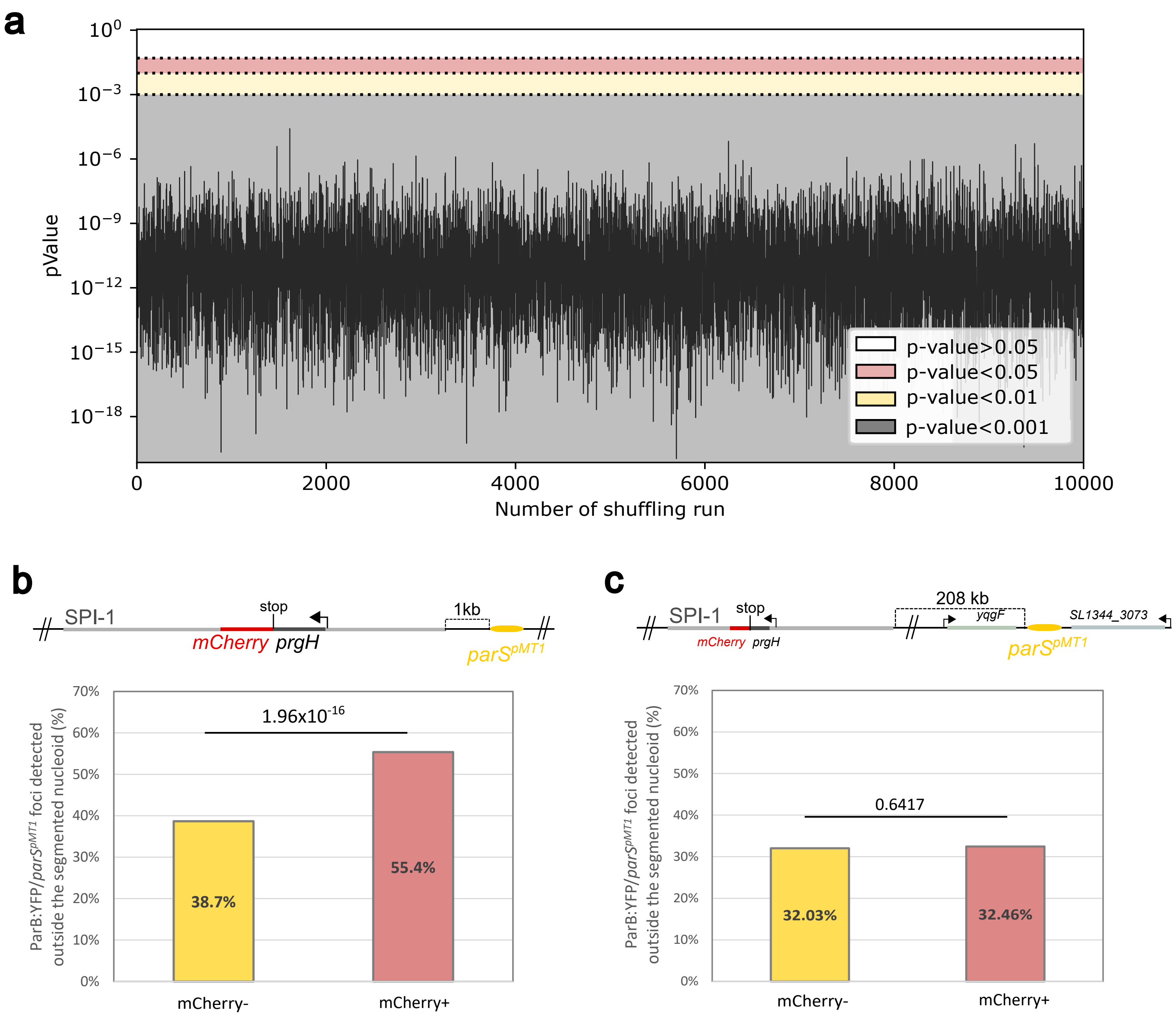
a
pValue
p-value>0.05
p-value<0.05
p-value<0.01
p-value<0.001
Number of shuffling run
b
SPI-1
stop
1kb
mCherry prgH
parSpMT1
ParB:YFP/parSpMT1 foci detected outside the segmented nucleoid (%)
1.96x10-16
38.7%
55.4%
mCherry-
mCherry+
c
SPI-1
stop
208 kb
yqgF
SL1344_3073
mCherry prgH
parSpMT1
ParB:YFP/parSpMT1 foci detected outside the segmented nucleoid (%)
0.6417
32.03%
32.46%
mCherry-
mCherry+

Supplementary Figure 9 ParB:YFP/*parS*^{pMT1} foci localization in *Salmonella* cells

a Computation of the *p*-value, obtained from a two-sided Mann-Whitney test applied to the ParB:YFP/*parS*^{pMT1} foci distances from the nucleoid surface in both mCherry$^-$ and mCherry$^+$. To avoid a side effect of the population size, we used the same number of foci for each population (n=652). To do so, the distances of all the mCherry$^+$ foci were compared to a random subpopulation of mCherry$^-$ foci (repeated 10,000 times). This approach demonstrates that disparities in population size do not influence the significance of the difference in localization between the two populations.

b Histogram representing the fraction of the ParB:YFP/*parS*^{pMT1} foci at SPI-1 locus detected outside the segmented nucleoid region, in both mCherry$^-$ and mCherry$^+$ populations grown in ESP condition. Significantly different conditions are marked with three stars. Statistical comparisons were performed using the two-sided Chi-square test and the p-value is shown in the plot. Number of foci analyzed are 5,858 (mCherry-) and 652 (mCherry+).

c Histogram representing the fraction of the ParB:YFP/*parS*^{pMT1} foci at *yqgF* detected outside the segmented nucleoid region, in both mCherry$^-$ and mCherry$^+$ populations grown in ESP. Statistical comparisons were performed using the two-sided Chi-square test and the p-value is shown in the plot. Number of foci analyzed are 11,463 (mCherry-) and 1,131 (mCherry+).

Supplementary Table 1: Main functions of SPIs encoded in S. Typhimurium SL1344

***Salmonella* Pathogenicity Island**	**Function**	**Co-regulated with SPI-1**	**Reference**
SPI-6	Encodes a type VI secretion system (T6SS) that enables gut colonization by killing commensal bacteria, it also contributes to intracellular replication and systemic dissemination in mice.	NR	[3,4]
SPI-16	Encodes genes responsible for O-antigen glucosylation, enhancing long-term intestinal persistence and fecal shedding of *Salmonella* in mice	NR	[5]
SPI-14	Encodes *loiA*, which regulates SPI-1 activation in low oxygen conditions, promoting *Salmonella* intestinal invasion.	Yes	[6]
SPI-5	Encodes effectors involved in different stages of infection: SopB, secreted by T3SS-1, promotes invasion, while PipB, translocated by T3SS-2, promotes intra-macrophage survival.	Yes	[7]
SPI-11	Encodes several genes that are critical for *Salmonella* survival within macrophages**.**	NR	[8,9]
SPI-2	Encodes for Type 3 secretion system (T3SS-2) and effectors essential for survival in macrophages.	Yes	[10]
SPI-12	Encodes *sspH2*, secreted by T3SS-2, which contributes to immune evasion and intracellular survival.	NR	[11]
SPI-9	SPI-9 in *S.* Typhimurium is not well characterized, but in *S. Typhi*, it encodes a Type 1 secretion system and an adhesin protein involved in epithelial cell adhesion under high osmolarity and low pH conditions.	NR	[12]

SPI-1	Encodes for Type 3 secretion system (T3SS-1) and effectors essential for invasion and cytosolic lifestyle.	NA	[13]
SPI-13	Encodes genes essential for virulence in mice and survival within macrophages.	NR	[14,15]
SPI-3	Encodes the *mgtCB* gene, a high-affinity Mg2+ uptake system required for *Salmonella* survival in macrophages and low Mg2+ environments. It also contains an adhesin essential for fibronectin binding and intestinal persistence.	NR	[16,17]
SPI-4	Encodes a type 1 secretion system and a major adhesin protein SiiE, which are important for bacterial entry into epithelial cells by promoting clustering and membrane ruffling at the invasion site.	Yes	[18,19]

NR: Not Reported in *S.* Typhimurium
NA: Not Applicable

Supplementary Table 2: SPI encoded genes upregulated in the GFP+ population

SPI	Gene name	Encoded Product	KEGG Orthology (KO)	KO sub category
SPI-5	*pipC*	Cell invasion protein	not assigned	not assigned
SPI-5	*sopB*	Type III secretion system effector protein	Human Diseases	Infectious disease: bacterial
SPI-11	*SL1344_1177*	Predicted bacteriophage protein	not assigned	not assigned
SPI-11	*pagC*	Outer membrane invasion protein (PagC)	Environmental Information Processing	Signal transduction
SPI-2	*ssrB*	Two-component system, LuxR family, secretion system response regulator SsrB	Environmental Information Processing	Signal transduction
SPI-1	*avrA*	Type III secretion system (T3SS) effector protein-regulator of *Salmonella*-induced inflammatory response	Human Diseases	Infectious disease: bacterial
SPI-1	*sprB*	AraC-family transcriptional regulator	not assigned	not assigned
SPI-1	*hilC*	AraC-family transcriptional regulator	not assigned	not assigned
SPI-1	*orgC*	Type III secretion system effector protein	not assigned	not assigned
SPI-1	*orgB*	Component of the sorting	not assigned	not assigned

		Platform of the T3SS1		
SPI-1	*orgA*	Component of the sorting Platform of the T3SS1	not assigned	not assigned
SPI-1	*prgK*	T3SS1 inner membrane ring protein	Brite Hierarchies	Protein families: signaling and cellular processes
SPI-1	*prgJ*	Component of the T3SS1	Human Diseases	Infectious disease: bacterial
SPI-1	*prgH*	Component of the T3SS1	Brite Hierarchies	Protein families: signaling and cellular processes
SPI-1	*hilD*	AraC-family transcriptional regulator. SPI-1 master regulator	not assigned	not assigned
SPI-1	*hilA*	Transcriptional regulator HilA, main transcriptional regulator of SPI1	Brite Hierarchies	Protein families: genetic information processing
SPI-1	*sptP*	T3SS1 effector protein	Human Diseases	Infectious disease: bacterial
SPI-1	*sicP*	Chaperone (associated with virulence)	Not assigned	not assigned
SPI-1	*iacP*	Required for the invasion of nonphagocytic cells.	Metabolism	Lipid metabolism
SPI-1	*sipA*	T3SS1 effector protein-involved in actin bundling and polymerisation leading to epithelial cell invasion and formation of the SCV	Human Diseases	Infectious disease: bacterial

SPI-1	*sipD*	Part of the T3SS1 Translocon	Human Diseases	Infectious disease: bacterial
SPI-1	*sipC*	T3SS1 effector protein-involved in bacterial entry by actin bundling and part of the Translocon	Human Diseases	Infectious disease: bacterial
SPI-1	*sipB*	T3SS1 effector protein	Human Diseases	Infectious disease: bacterial
SPI-1	*sicA*	T3SS1 - associated chaperone	not assigned	not assigned
SPI-1	*spaS*	T3SS1 export apparatus switch protein	Brite Hierarchies	Protein families: signaling and cellular processes
SPI-1	*spaR*	T3SS1 export apparatus protein	Brite Hierarchies	Protein families: signaling and cellular processes
SPI-1	*spaQ*	T3SS1 export apparatus protein	Brite Hierarchies	Protein families: signaling and cellular processes
SPI-1	*spaP*	T3SS1 export apparatus protein	Brite Hierarchies	Protein families: signaling and cellular processes
SPI-1	*spaO*	Component of the sorting Platform of the T3SS1	Environmental Information Processing	Membrane transport
SPI-1	*invJ*	T3SS1 protein	Brite Hierarchies	Protein families: signaling and cellular processes
SPI-1	*invC*	component of the T3SS1 sorting platform	Brite Hierarchies	Protein families: signaling and cellular processes
SPI-1	*invB*	T3SS1 chaperone	Brite Hierarchies	Protein families: signaling and cellular processes
SPI-1	*invE*	T3SS1 protein	Brite Hierarchies	Protein families: signaling and cellular processes
SPI-1	*invG*	T3SS1 outer membrane ring protein	Brite Hierarchies	Protein families: signaling and cellular processes

SPI-1	*InvR*	small RNA	not assigned	not assigned
SPI-3	*cigR*	hypothetical inner membrane protein	not assigned	not assigned
SPI-4	*siiB*	inner membrane protein	not assigned	not assigned
SPI-4	*siiC*	Component of a type-I secretion system 4 (T1SS4)	Brite Hierarchies	Protein families: signaling and cellular processes
SPI-4	*siiD*	Component of T1SS4	Brite Hierarchies	Protein families: signaling and cellular processes

Supplementary Table 3. Strains used in this study

Name	Description	Source or Reference
Wild-Type	*Salmonella enterica* serovar Typhimurium SL1344	20
MKB1	P_{prgH}-*gfp cm*^{R}	[20]
MKB7	*hilE*::*cm*^{R}	This study
MKB13	*hns-3xFLAG cm*^{R}	This study
MKB15	*hilE* P_{prgH}-*gfp cm*^{R}	This study
MKB16	*hilD*::*cm*^{R}	This study
MKB17	*rpoC-3xFLAG cm*^{R}	This study
MKB18	*hilD-3xFLAG* cm^R	This study
MKB33	*prgH(stop)mCherry parS*^{pMT1}*cm*^{R}	This study
MKB49	*prgH(stop)mCherry yqgf::parS*^{pMT1}*cm*^{R}	This study

Supplementary Table 4. Plasmids used in this study

Name	Description	Source or Reference
pKD3	PCR template plasmid containing a chloramphenicol resistance cassette.	[21]
pKD4	PCR template plasmid containing a kanamycin resistance cassette.	[21]
pKD46	Plasmid encoding λ-Red system for recombination.	[21]
pCP20	Plasmid encoding FLP recombinase.	[21]
pGBKD3-parSPMT1	PCR template plasmid containing parSPMT1 CmR cassette	[22]
pMKB1	PCR template plasmid containing promoter less mCherry.	This study
pFPV25.1	Template plasmid carrying a promoterless gfp gene, used as backbone for constructing pMKB1.	[23] -gift from Raphael Valdivia (Addgene plasmid # 20668 ; http://n2t.net/addgene:20668 ; RRID:Addgene_20668)
pFCcGi	Source of the promoterless mCherry gene used to replace gfp in pFPV25.	[24] -gift from Sophie Helaine & David Holden (Addgene plasmid # 59324 ;

		http://n2t.net/addgene:59324 ; RRID:Addgene_59324)
pSPB5	P_{lac} *parBPMT1-yfp* amp^R	This study
pFH2973	CFP-Δ30ParBP1/ YFP-Δ23ParBT1 expression vector	[25]

Supplementary Table 5. Hi-C libraries performed in this study

Id	Library	Conditions	Total pairs processed	Valid interactions
MKL3	WT EEP rep1	growth in LB at 37°C EEP (OD 0.1)	6273869	5359451
MKL8	WT EEP rep2		9180949	8922179
MKL19	WT EEP rep3		10830147	6330485
WT EEP merged	**WT EEP merge**		26336107	**20 649 013**
MKL1	WT ESP rep1	growth in LB at 37°C ESP (OD 2)	7837734	7154256
MKL4	WT ESP rep2		6893876	6688347
MKL9	WT ESP rep3		8973183	5648927
MKL20	WT ESP rep3		11064794	9626700
WT ESP merged	**WT ESP merge**		34663755	**29 024 094**
MKL15	WT Anaerobic growth	Anaerobic growth in LB at 37°C to OD 0.3	4077901	3 999 140
MKL15_bis*			1299562	1 292 701
MKL15_Merged	**WT Anaerobic growth merged**		5379716	**5 263 346**
MKL16	WT Oxygen shock	Static growth in LB at 37°C to OD 0.3 then 15 min aerobic growth	2802296	2 754 855
MKL16_bis*			3544955	3 515 850
MKL16_Merged	**WT Oxygen shock merged**		6344054	**6 232 640**
MKL41	**SL1344 *hilE* P_{prgH}-*gfp* ESP GFP+**	growth in LB at 37°C ESP (OD 2) Sorted only GFP positive population	6555694	**5 815 621**
MKL42	**SL1344 *hilE* P_{prgH}-*gfp* ESP GFP-**	growth in LB at 37°C ESP (OD 2) Sorted only GFP negative population	5921230	**5 622 714**
MKL72	SL1344 *hilD* ESP rep1	growth in LB at 37°C ESP (OD 2)	7282238	6 956 139
MKL134	SL1344 *hilD* ESP rep2		6191117	5 883 056
DhilD_ESP_merged	**SL1344 *hilD* ESP merged**		13475614	**12 839 983**

MKL81	SL1344 P_{prgH}-*gfp* ESP GFP+	growth in LB at 37°C ESP (OD 2) Sorted only GFP positive population	2271159	2 135 781
MKL81_bis*			10432522	8 350 519
MKL81_Merged	**SL1344 P_{prgH}-*gfp* ESP GFP+ Merged**		12703915	**9 758 492**
MKL82	SL1344 P_{prgH}-*gfp* ESP GFP-	growth in LB at 37°C ESP (OD 2) Sorted only GFP negative population	1973214	1 920 635
MKL82_bis*			9032070	8 403 073
MKL82_Merged	**SL1344 P_{prgH}-*gfp* ESP GFP-**		11005464	**10 109 980**

*Libraries were re-sequenced to have higher valid reads number.

Supplementary Table 6. ChIP-seq libraries performed in this study

ID	Library	Conditions	Mapped_reads_count
MKL91	Input SL1344 *hilD*-3xFLAG EEP rep1	growth in LB at 37°C EEP (OD 0.1)	36194599
MKL93	Input SL1344 *hilD*-3xFLAG EEP rep2		31646346
**merge**	**Input SL1344 *hilD*-3xFLAG EEP merge**		**67 840 945**
MKL92	Input SL1344 *hilD*-3xFLAG ESP rep1	growth in LB at 37°C ESP (OD 2)	36937073
MKL94	Input SL1344 *hilD*-3xFLAG ESP rep2		29872125
merge	**Input SL1344 *hilD*-3xFLAG ESP merge**		**66 809 198**
MKL95	IP SL1344 *hilD*-3xFLAG EEP rep1	growth in LB at 37°C EEP (OD 0.1)	11138115
MKL97	IP SL1344 *hilD*-3xFLAG EEP rep2		10560086
merge	**IP SL1344 *hilD*-3xFLAG EEP merge**		**21 698 201**
MKL96	IP SL1344 *hilD*-3xFLAG ESP rep1	growth in LB at 37°C ESP (OD 2)	14354457

MKL98	IP SL1344 *hilD*-3xFLAG ESP rep2		9670734
	**IP SL1344 *hilD*-3xFLAG ESP merge**		**24 025 191**
MKL99	Input SL1344 *rpoC*-3xFLAG EEP rep1	growth in LB at 37°C EEP (OD 0.1)	29246161
MKL101	Input SL1344 *rpoC*-3xFLAG EEP rep2		26394520
MKL135	Input SL1344 *rpoC*-3xFLAG EEP rep3		20486978
MKL137	Input SL1344 *rpoC*-3xFLAG EEP rep4		23789930
**merge**	**Input SL1344 *rpoC*-3xFLAG EEP merge**		**99 917 589**
MKL103	IP SL1344 *rpoC*-3xFLAG EEP rep1	growth in LB at 37°C EEP (OD 0.1)	20969598
MKL105	IP SL1344 *rpoC*-3xFLAG EEP rep2		15991264
MKL139	IP SL1344 *rpoC*-3xFLAG EEP rep3		15894320
MKL141	IP SL1344 *rpoC*-3xFLAG EEP rep4		16582489
**merge**	**IP SL1344 *rpoC*-3xFLAG EEP merge**		**69 437 671**
MKL107	Input SL1344 *hns*-3xFLAG EEP rep1	growth in LB at 37°C EEP (OD 0.1)	31922981
MKL109	Input SL1344 *hns*-3xFLAG EEP rep2		27133135
**merge**	**Input SL1344 *hns*-3xFLAG EEP merge**		**59 056 116**

MKL108	Input SL1344 *hns*-3xFLAG ESP rep1	growth in LB at 37°C ESP (OD 2)	20217682
MKL110	Input SL1344 *hns*-3xFLAG ESP rep2		25978731
**merge**	**Input SL1344 *hns*-3xFLAG ESP merge**		**46 196 413**
MKL111	IP SL1344 hns-3xFLAG EEP rep1	growth in LB at 37°C EEP (OD 0.1)	25621825
MKL113	IP SL1344 hns-3xFLAG EEP rep2		28369797
**merge**	**IP SL1344 hns-3xFLAG EEP merge**		**53 991 622**
MKL112	IP SL1344 hns-3xFLAG ESP rep1	growth in LB at 37°C ESP (OD 2)	18856253
MKL114	IP SL1344 hns-3xFLAG ESP rep2		30330205
**merge**	**IP SL1344 hns-3xFLAG ESP merge**		**49 186 458**
MKL115	Input SL1344 hns-3xFLAG P_{prgH}-*gfp* ESP GFP+ rep1	growth in LB at 37°C ESP (OD 2) Sorted only GFP positive population	33930348
MKL117	Input SL1344 hns-3xFLAG P_{prgH}-*gfp* ESP GFP+ rep2		29563335
**merge**	**Input SL1344 hns-3xFLAG P_{prgH}-*gfp* ESP GFP+ merge**		**63 493 683**
MKL116	Input SL1344 hns-3xFLAG P_{prgH}-*gfp* ESP GFP- rep1	growth in LB at 37°C ESP (OD 2) Sorted only GFP negative population	38986395
MKL118	Input SL1344 hns-3xFLAG		36089550

	P_{prgH}-*gfp* ESP GFP- rep2		
**merge**	**Input SL1344 hns-3xFLAG** $\boldsymbol{P_{prgH}}$**-*gfp* ESP GFP- merge**		**75 075 945**
MKL119	IP SL1344 hns-3xFLAG P_{prgH}-*gfp* ESP GFP+ rep1	growth in LB at 37°C ESP (OD 2) Sorted only GFP positive population	56103616
MKL121	IP SL1344 hns-3xFLAG P_{prgH}-*gfp* ESP GFP+ rep2		32316035
**merge**	**IP SL1344 hns-3xFLAG** $\boldsymbol{P_{prgH}}$**-*gfp* ESP GFP+ merge**		**88 419 651**
MKL120	IP SL1344 hns-3xFLAG P_{prgH}-*gfp* ESP GFP- rep1	growth in LB at 37°C ESP (OD 2) Sorted only GFP negative population	45910688
MKL122	IP SL1344 hns-3xFLAG P_{prgH}-*gfp* ESP GFP- rep2		67132426
**merge**	**IP SL1344 hns-3xFLAG** $\boldsymbol{P_{prgH}}$**-*gfp* ESP GFP- merge**		**113 043 114**

Supplementary Table 7. RNA-seq libraries performed in this study

ID	Library	Conditions	Total reads	Total mapped reads
MKR21	Total RNA, SL1344 P_{prgH}-*gfp* rep1	growth in LB at 37°C ESP (OD 2), sorted, GFP^{+} population	34 852 819	13 648 886
MKR23	Total RNA, SL1344 P_{prgH}-*gfp* rep2		43 388 497	19 678 566
MKR22	Total RNA, SL1344 P_{prgH}-*gfp* rep1	growth in LB at 37°C ESP (OD 2),sorted, GFP^{-} population	37 839 496	12 973 695
MKR24	Total RNA, SL1344 P_{prgH}-*gfp* rep2		28 128 599	11 989 278

References

1. Kröger, C. *et al.* An infection-relevant transcriptomic compendium for Salmonella enterica Serovar Typhimurium. *Cell Host Microbe* **14**, 683–695 (2013).
2. Lioy, V. S., Junier, I., Lagage, V., Vallet, I. & Boccard, F. Distinct Activities of Bacterial Condensins for Chromosome Management in Pseudomonas aeruginosa. *Cell Rep.* **33**, 108344 (2020).
3. Mulder, D. T., Cooper, C. A. & Coombes, B. K. Type VI secretion system-associated gene clusters contribute to pathogenesis of Salmonella enterica serovar Typhimurium. *Infect. Immun.* **80**, 1996–2007 (2012).
4. Sana, T. G. *et al.* Salmonella Typhimurium utilizes a T6SS-mediated antibacterial weapon to establish in the host gut. *Proc. Natl. Acad. Sci. U. S. A.* **113**, E5044-5051 (2016).
5. Bogomolnaya, L. M., Santiviago, C. A., Yang, H.-J., Baumler, A. J. & Andrews-Polymenis, H. L. 'Form variation' of the O12 antigen is critical for persistence of Salmonella Typhimurium in the murine intestine. *Mol. Microbiol.* **70**, 1105–1119 (2008).
6. Jiang, L. *et al.* Signal transduction pathway mediated by the novel regulator LoiA for low oxygen tension induced Salmonella Typhimurium invasion. *PLoS Pathog.* **13**, e1006429 (2017).

7. Knodler, L. A. *et al.* Salmonella effectors within a single pathogenicity island are differentially expressed and translocated by separate type III secretion systems. *Mol. Microbiol.* **43**, 1089–1103 (2002).
8. Miller, S. I., Kukral, A. M. & Mekalanos, J. J. A two-component regulatory system (phoP phoQ) controls Salmonella typhimurium virulence. *Proc. Natl. Acad. Sci. U. S. A.* **86**, 5054–5058 (1989).
9. Lee, Y. H., Kim, S., Helmann, J. D., Kim, B.-H. & Park, Y. K. RaoN, a small RNA encoded within Salmonella pathogenicity island-11, confers resistance to macrophage-induced stress. *Microbiol. Read. Engl.* **159**, 1366–1378 (2013).
10. Hensel, M. *et al.* Genes encoding putative effector proteins of the type III secretion system of Salmonella pathogenicity island 2 are required for bacterial virulence and proliferation in macrophages. *Mol. Microbiol.* **30**, 163–174 (1998).
11. McGhie, E. J., Brawn, L. C., Hume, P. J., Humphreys, D. & Koronakis, V. Salmonella takes control: effector-driven manipulation of the host. *Curr. Opin. Microbiol.* **12**, 117–124 (2009).
12. Velásquez, J. C. *et al.* SPI-9 of Salmonella enterica serovar Typhi is constituted by an operon positively regulated by RpoS and contributes to adherence to epithelial cells in culture. *Microbiology* **162**, 1367–1378 (2016).
13. Raffatellu, M. *et al.* SipA, SopA, SopB, SopD, and SopE2 contribute to Salmonella enterica serotype typhimurium invasion of epithelial cells. *Infect. Immun.* **73**, 146–154 (2005).
14. Shi, L. *et al.* Proteomic analysis of Salmonella enterica serovar typhimurium isolated from RAW 264.7 macrophages: identification of a novel protein that contributes to the replication of serovar typhimurium inside macrophages. *J. Biol. Chem.* **281**, 29131–29140 (2006).
15. Haneda, T., Ishii, Y., Danbara, H. & Okada, N. Genome-wide identification of novel genomic islands that contribute to Salmonella virulence in mouse systemic infection. *FEMS Microbiol. Lett.* **297**, 241–249 (2009).
16. Blanc-Potard, A. B. & Groisman, E. A. The Salmonella selC locus contains a pathogenicity island mediating intramacrophage survival. *EMBO J.* **16**, 5376–5385 (1997).
17. Blanc-Potard, A. B., Solomon, F., Kayser, J. & Groisman, E. A. The SPI-3 pathogenicity island of Salmonella enterica. *J. Bacteriol.* **181**, 998–1004 (1999).
18. Morgan, E., Bowen, A. J., Carnell, S. C., Wallis, T. S. & Stevens, M. P. SiiE is secreted by the Salmonella enterica serovar Typhimurium pathogenicity island 4-encoded secretion system and contributes to intestinal colonization in cattle. *Infect. Immun.* **75**, 1524–1533 (2007).
19. Lorkowski, M., Felipe-López, A., Danzer, C. A., Hansmeier, N. & Hensel, M. Salmonella enterica invasion of polarized epithelial cells is a highly cooperative effort. *Infect. Immun.* **82**, 2657–2667 (2014).

20. Hautefort, I., Proença, M. J. & Hinton, J. C. D. Single-Copy Green Fluorescent Protein Gene Fusions Allow Accurate Measurement of Salmonella Gene Expression In Vitro and during Infection of Mammalian Cells. *Appl. Environ. Microbiol.* **69**, 7480–7491 (2003).
21. Datsenko, K. A. & Wanner, B. L. One-step inactivation of chromosomal genes in Escherichia coli K-12 using PCR products. *Proc. Natl. Acad. Sci.* **97**, 6640–6645 (2000).
22. Espeli, O., Mercier, R. & Boccard, F. DNA dynamics vary according to macrodomain topography in the E. coli chromosome. *Mol. Microbiol.* **68**, 1418–1427 (2008).
23. Valdivia, R. H. & Falkow, S. Bacterial genetics by flow cytometry: rapid isolation of Salmonella typhimurium acid-inducible promoters by differential fluorescence induction. *Mol. Microbiol.* **22**, 367–378 (1996).
24. Figueira, R., Watson, K. G., Holden, D. W. & Helaine, S. Identification of Salmonella Pathogenicity Island-2 Type III Secretion System Effectors Involved in Intramacrophage Replication of S. enterica Serovar Typhimurium: Implications for Rational Vaccine Design. *mBio* **4**, 10.1128/mbio.00065-13 (2013).
25. Nielsen, H. J., Ottesen, J. R., Youngren, B., Austin, S. J. & Hansen, F. G. The Escherichia coli chromosome is organized with the left and right chromosome arms in separate cell halves. *Mol. Microbiol.* **62**, 331–338 (2006).
