# Supplementary material for: Bacterial chromatin remodeling associated with transcription-induced domains at pathogenicity Islands
Source: Nat Commun. 2026 Jan 8;17:161. doi: 10.1038/s41467-025-67746-w (PMC12783615; doi:10.1038/s41467-025-67746-w)
Supplement: Supplementary file 2 — Description of Additional Supplementary Files [file 41467_2025_67746_MOESM2_ESM.pdf]

Description of Additional Supplementary Files

Supplementary Data 1: RNA-seq analysis performed in this study

Supplementary Data 2: MACS2 analyses of H-NS ChIP-seq performed in sorted GFP+/GFP- populations of Salmonella.

Supplementary Data 3: pValues for Figure 3

Supplementary Data 4: pValues for Figure 4B

Supplementary Data 5: statistical report of the RNA-seq in sorted populations of *Salmonella*

Supplementary Data 6: statistical report of the RNA-seq in sorted populations of *Salmonella* binned at 1kb.
