# Supplementary material for: Bacterial chromatin remodeling associated with transcription-induced domains at pathogenicity Islands
Source: Nat Commun. 2026 Jan 8;17:161. doi: 10.1038/s41467-025-67746-w (PMC12783615; doi:10.1038/s41467-025-67746-w)
Supplement: Supplementary file 6 — Supplementary Data 4 [file 41467_2025_67746_MOESM6_ESM.pdf]

```
##Stats in HNS occupancy

## R script

# @author: Vicky LIOY

library(tidyr)

library(readxl)

library(dplyr)

library(openxlsx)

library(Cairo)

library(ggplot2)


#Load the table with data:


HNS_neg_SPIs_chr1<-read_excel("HNS_neg_SPIs.chr1.xlsx")

HNS_pos_SPIs_chr1<-read_excel("HNS_pos_SPIs.chr1.xlsx")


###convert to dataframe:

my_data_neg<-as.data.frame(HNS_neg_SPIs_chr1)

my_data_pos<-as.data.frame(HNS_pos_SPIs_chr1)


####Select the data to compare:


HNS_pos_SPIs_chr1_stat <- HNS_pos_SPIs_chr1 %>%

  select(bin_start, Id, ChIP)


HNS_neg_SPIs_chr1_stat <- HNS_neg_SPIs_chr1 %>%

  select(bin_start, Id, ChIP)


#subset SPIs:


SPI5_neg <- subset(HNS_neg_SPIs_chr1_stat, Id == 'SPI-5')

SPI1_neg<- subset(HNS_neg_SPIs_chr1_stat, Id == 'SPI-1')
```

```
SPI2_neg <- subset(HNS_neg_SPIs_chr1_stat, Id == 'SPI-2')
SPI3_neg <- subset(HNS_neg_SPIs_chr1_stat, Id == 'SPI-3')
SPI6_neg <- subset(HNS_neg_SPIs_chr1_stat, Id == 'SPI-6')
SPI16_neg <- subset(HNS_neg_SPIs_chr1_stat, Id == 'SPI-16')
SPI14_neg <- subset(HNS_neg_SPIs_chr1_stat, Id == 'SPI-14')
SPI11_neg<- subset(HNS_neg_SPIs_chr1_stat, Id == 'SPI-11')
SPI12_neg <- subset(HNS_neg_SPIs_chr1_stat, Id == 'SPI-12+oaf')
SPI13_neg <- subset(HNS_neg_SPIs_chr1_stat, Id == 'SPI-13')
SPI4_neg <- subset(HNS_neg_SPIs_chr1_stat, Id == 'SPI-4')
SPI9_neg<- subset(HNS_neg_SPIs_chr1_stat, Id == 'SPI-9')
Other_neg<- subset(HNS_neg_SPIs_chr1_stat, Id == 'Other')

SPI5_pos <- subset(HNS_pos_SPIs_chr1_stat, Id == 'SPI-5')
SPI1_pos<- subset(HNS_pos_SPIs_chr1_stat, Id == 'SPI-1')
SPI2_pos <- subset(HNS_pos_SPIs_chr1_stat, Id == 'SPI-2')
SPI3_pos <- subset(HNS_pos_SPIs_chr1_stat, Id == 'SPI-3')
SPI6_pos <- subset(HNS_pos_SPIs_chr1_stat, Id == 'SPI-6')
SPI16_pos <- subset(HNS_pos_SPIs_chr1_stat, Id == 'SPI-16')
SPI14_pos <- subset(HNS_pos_SPIs_chr1_stat, Id == 'SPI-14')
SPI11_pos<- subset(HNS_pos_SPIs_chr1_stat, Id == 'SPI-11')
SPI12_pos <- subset(HNS_pos_SPIs_chr1_stat, Id == 'SPI-12+oaf')
SPI13_pos <- subset(HNS_pos_SPIs_chr1_stat, Id == 'SPI-13')
SPI4_pos <- subset(HNS_pos_SPIs_chr1_stat, Id == 'SPI-4')
SPI9_pos<- subset(HNS_pos_SPIs_chr1_stat, Id == 'SPI-9')
Other_pos<- subset(HNS_pos_SPIs_chr1_stat, Id == 'Other')

#combine the SPIs neg and pos:

SPI5_HNS <- data.frame(SPI5neg = SPI5_neg$ChIP, SPI5pos = SPI5_pos$ChIP)
SPI1_HNS <- data.frame(SPI1neg = SPI1_neg$ChIP, SPI1pos = SPI1_pos$ChIP)
SPI2_HNS <- data.frame(SPI2neg = SPI2_neg$ChIP, SPI2pos = SPI2_pos$ChIP)
```

```
SPI3_HNS <- data.frame(SPI3neg = SPI3_neg$ChIP, SPI3pos = SPI3_pos$ChIP)

SPI4_HNS <- data.frame(SPI4neg = SPI4_neg$ChIP, SPI4pos = SPI4_pos$ChIP)

SPI6_HNS <- data.frame(SPI6neg = SPI6_neg$ChIP, SPI6pos = SPI6_pos$ChIP)

SPI16_HNS <- data.frame(SPI16neg = SPI16_neg$ChIP, SPI16pos = SPI16_pos$ChIP)

SPI14_HNS <- data.frame(SPI14neg = SPI14_neg$ChIP, SPI14pos = SPI14_pos$ChIP)

SPI11_HNS <- data.frame(SPI11neg = SPI11_neg$ChIP, SPI11pos = SPI11_pos$ChIP)

SPI12_HNS <- data.frame(SPI12neg = SPI12_neg$ChIP, SPI12pos = SPI12_pos$ChIP)

SPI13_HNS <- data.frame(SPI13neg = SPI13_neg$ChIP, SPI13pos = SPI13_pos$ChIP)

SPI9_HNS <- data.frame(SPI9neg = SPI9_neg$ChIP, SPI9pos = SPI9_pos$ChIP)

Other_HNS <- data.frame(Otherneg = Other_neg$ChIP, Otherpos = Other_pos$ChIP)


#Perform stats to comapre if they are significantly different:

library(ggpubr)


#SPI-5

wilcox.test(SPI5_HNS$'SPI5neg', SPI5_HNS$'SPI5pos')

Wilcoxon rank sum exact test

data:  SPI5_HNS$SPI5neg and SPI5_HNS$SPI5pos

W = 62, p-value = 0.393

alternative hypothesis: true location shift is not equal to 0


#SPI-1

wilcox.test(SPI1_HNS$'SPI1neg', SPI1_HNS$'SPI1pos')

Wilcoxon rank sum exact test

data:  SPI1_HNS$SPI1neg and SPI1_HNS$SPI1pos

W = 1358, p-value = 0.0001338

alternative hypothesis: true location shift is not equal to 0


#SPI-2


wilcox.test(SPI2_HNS$'SPI2neg', SPI2_HNS$'SPI2pos')
```

Wilcoxon rank sum exact test

data: SPI2_HNS$SPI2neg and SPI2_HNS$SPI2pos

W = 866, p-value = 0.5305

alternative hypothesis: true location shift is not equal to 0

#SPI-3

wilcox.test(SPI3_HNS$'SPI3neg', SPI3_HNS$'SPI3pos')

Wilcoxon rank sum exact test

data: SPI3_HNS$SPI3neg and SPI3_HNS$SPI3pos

W = 149, p-value = 0.8919

alternative hypothesis: true location shift is not equal to 0

#SPI-4

wilcox.test(SPI4_HNS$'SPI4neg', SPI4_HNS$'SPI4pos')

Wilcoxon rank sum exact test

data: SPI4_HNS$SPI4neg and SPI4_HNS$SPI4pos

W = 247, p-value = 0.9167

alternative hypothesis: true location shift is not equal to 0

#SPI-6

wilcox.test(SPI6_HNS$'SPI6neg', SPI6_HNS$'SPI6pos')

Wilcoxon rank sum exact test

data: SPI6_HNS$SPI6neg and SPI6_HNS$SPI6pos

W = 1032, p-value = 0.5878

alternative hypothesis: true location shift is not equal to 0

#SPI16

wilcox.test(SPI16_HNS$'SPI16neg', SPI16_HNS$'SPI16pos')

Wilcoxon rank sum exact test

data: SPI16_HNS$SPI16neg and SPI16_HNS$SPI16pos

W = 12, p-value = 1

alternative hypothesis: true location shift is not equal to 0

#SPI14

wilcox.test(SPI14_HNS$'SPI14neg', SPI14_HNS$'SPI14pos')

Wilcoxon rank sum exact test

data: SPI14_HNS$SPI14neg and SPI14_HNS$SPI14pos

W = 33, p-value = 0.9591

alternative hypothesis: true location shift is not equal to 0

#SPI11

wilcox.test(SPI11_HNS$'SPI11neg', SPI11_HNS$'SPI11pos')

Wilcoxon rank sum exact test

data: SPI11_HNS$SPI11neg and SPI11_HNS$SPI11pos

W = 41, p-value = 0.3823

alternative hypothesis: true location shift is not equal to 0

#SPI12

wilcox.test(SPI12_HNS$'SPI12neg', SPI12_HNS$'SPI12pos')

Wilcoxon rank sum exact test

data: SPI12_HNS$SPI12neg and SPI12_HNS$SPI12pos

W = 130, p-value = 0.9556

alternative hypothesis: true location shift is not equal to 0

#SPI13

wilcox.test(SPI13_HNS$'SPI13neg', SPI13_HNS$'SPI13pos')

Wilcoxon rank sum exact test

data: SPI13_HNS$SPI13neg and SPI13_HNS$SPI13pos

W = 39, p-value = 0.5054

alternative hypothesis: true location shift is not equal to 0

#SPI9

wilcox.test(SPI9_HNS$'SPI9neg', SPI9_HNS$'SPI9pos')

Wilcoxon rank sum exact test

data: SPI9_HNS$SPI9neg and SPI9_HNS$SPI9pos

W = 93, p-value = 0.07886

alternative hypothesis: true location shift is not equal to 0

#Other

wilcox.test(Other_HNS$'Otherneg', Other_HNS$'Otherpos')

Wilcoxon rank sum test with continuity correction

data: Other_HNS$Otherneg and Other_HNS$Otherpos

W = 9188664, p-value < 2.2e-16

alternative hypothesis: true location shift is not equal to 0
