# Supplementary material for: Bacterial chromatin remodeling associated with transcription-induced domains at pathogenicity Islands
Source: Nat Commun. 2026 Jan 8;17:161. doi: 10.1038/s41467-025-67746-w (PMC12783615; doi:10.1038/s41467-025-67746-w)
Supplement: Supplementary file 9 — Reporting Summary [file 41467_2025_67746_MOESM9_ESM.pdf]

nature portfolio

Corresponding author(s): Virginia Lioy

Last updated by author(s): Dec 2, 2025

Reporting Summary

Nature Portfolio wishes to improve the reproducibility of the work that we publish. This form provides structure for consistency and transparency in reporting. For further information on Nature Portfolio policies, see our Editorial Policies and the Editorial Policy Checklist.

Statistics

For all statistical analyses, confirm that the following items are present in the figure legend, table legend, main text, or Methods section.

n/a	Confirmed	
☐	☒	The exact sample size (*n*) for each experimental group/condition, given as a discrete number and unit of measurement
☐	☒	A statement on whether measurements were taken from distinct samples or whether the same sample was measured repeatedly
☐	☒	The statistical test(s) used AND whether they are one- or two-sided<br>*Only common tests should be described solely by name; describe more complex techniques in the Methods section.*
☒	☐	A description of all covariates tested
☒	☐	A description of any assumptions or corrections, such as tests of normality and adjustment for multiple comparisons
☐	☒	A full description of the statistical parameters including central tendency (e.g. means) or other basic estimates (e.g. regression coefficient) AND variation (e.g. standard deviation) or associated estimates of uncertainty (e.g. confidence intervals)
☐	☒	For null hypothesis testing, the test statistic (e.g. *F*, *t*, *r*) with confidence intervals, effect sizes, degrees of freedom and *P* value noted<br>*Give P values as exact values whenever suitable.*
☒	☐	For Bayesian analysis, information on the choice of priors and Markov chain Monte Carlo settings
☒	☐	For hierarchical and complex designs, identification of the appropriate level for tests and full reporting of outcomes
☒	☐	Estimates of effect sizes (e.g. Cohen's *d*, Pearson's *r*), indicating how they were calculated

Our web collection on statistics for biologists contains articles on many of the points above.

Software and code

Policy information about availability of computer code

Data collection	The datasets generated during this study have been deposited in the NCBI Gene Expression Omnibus (GEO, https://www.ncbi.nlm.nih.gov/geo/) under the accession number GSE289504. At this link a table describes each biological sample and data processing.<br>https://www.ncbi.nlm.nih.gov/geo/query/acc.cgi?acc=GSE289504
Data analysis	Github link to the scripts used for data analysis:<br>- RNAseq analysis: https://github.com/PF2-pasteur-fr/SARTools<br>- Hi-C analysis: https://github.com/js2264/OHCA<br>-ChIP-seq: https://deeptools.readthedocs.io/en/latest/index.html<br>-3DSIM: https://github.com/Rom-LB/SPI1<br>- mapping: Bowtie2 (v 2.4.2)<br>-peak calling: MACS2

For manuscripts utilizing custom algorithms or software that are central to the research but not yet described in published literature, software must be made available to editors and reviewers. We strongly encourage code deposition in a community repository (e.g. GitHub). See the Nature Portfolio guidelines for submitting code & software for further information.

Data

Policy information about availability of data

All manuscripts must include a data availability statement. This statement should provide the following information, where applicable:

- Accession codes, unique identifiers, or web links for publicly available datasets
- A description of any restrictions on data availability
- For clinical datasets or third party data, please ensure that the statement adheres to our policy

The ChIP-seq, RNA-seq and Hi-C data generated during in this study have been deposited in the NCBI Gene Expression Omnibus (GEO, https://www.ncbi.nlm.nih.gov/geo/) under the accession code GSE289504. (https://www.ncbi.nlm.nih.gov/geo/query/acc.cgi?acc=GSE289504)

Research involving human participants, their data, or biological material

Policy information about studies with human participants or human data. See also policy information about sex, gender (identity/presentation), and sexual orientation and race, ethnicity and racism.

Reporting on sex and gender	n/a
Reporting on race, ethnicity, or other socially relevant groupings	n/a
Population characteristics	n/a
Recruitment	n/a
Ethics oversight	n/a

Note that full information on the approval of the study protocol must also be provided in the manuscript.

Field-specific reporting

Please select the one below that is the best fit for your research. If you are not sure, read the appropriate sections before making your selection.

☒ Life sciences ☐ Behavioural & social sciences ☐ Ecological, evolutionary & environmental sciences

For a reference copy of the document with all sections, see nature.com/documents/nr-reporting-summary-flat.pdf

Life sciences study design

All studies must disclose on these points even when the disclosure is negative.

Sample size	For the RNA-seq, two independent experiments were carried out for each studied condition. For the Hi-C, one texperiment was performed for sorted data, while one to four replicates were done for heterogeneous cultures. For Chip-seq, see below.
Data exclusions	No data exclusion. As the different samples were grouped according to the biological conditions, they were all considered relevant.
Replication	Samples that passed quality controls were considered as replicates and used for anlaysis.
Randomization	Experiments were performed independently (on different days, with different medium preparation).
Blinding	n/a

Reporting for specific materials, systems and methods

We require information from authors about some types of materials, experimental systems and methods used in many studies. Here, indicate whether each material, system or method listed is relevant to your study. If you are not sure if a list item applies to your research, read the appropriate section before selecting a response.

Materials & experimental systems

n/a	Involved in the study
☒	☐ Antibodies
☒	☐ Eukaryotic cell lines
☒	☐ Palaeontology and archaeology
☒	☐ Animals and other organisms
☒	☐ Clinical data
☒	☐ Dual use research of concern
☒	☐ Plants

Methods

n/a	Involved in the study
☐	☒ ChIP-seq
☐	☒ Flow cytometry
☒	☐ MRI-based neuroimaging

Plants

Seed stocks	n/a
Novel plant genotypes	n/a
Authentication	n/a

ChIP-seq

Data deposition

☒ Confirm that both raw and final processed data have been deposited in a public database such as GEO.

☐ Confirm that you have deposited or provided access to graph files (e.g. BED files) for the called peaks.

Data access links<br>*May remain private before publication.*	https://www.ncbi.nlm.nih.gov/geo/query/acc.cgi?acc=GSE289504
Files in database submission	*Provide a list of all files available in the database submission.*
Genome browser session<br>(e.g. UCSC)	*Provide a link to an anonymized genome browser session for "Initial submission" and "Revised version" documents only, to enable peer review. Write "no longer applicable" for "Final submission" documents.*

Methodology

Replicates	ChIP-seq experiments were performed in duplicates or quadruplates
Sequencing depth	>200 coverage
Antibodies	ANTI-FLAG M2 affinity gel (Sigma-Aldrich, Cat# A2220)
Peak calling parameters	example of typical command: macs2 callpeak -t ./IP_HNS.bam -c ./Input_HNS.bam --name HNS_narrow_0.0005 --bdg --nomodel -f BAMPE --nolambda --extsize 300 -g 5.06e6 -q 0.0005 --outdir ./MACS2_output_HNS_narrow_0.0005
Data quality	Q>30
Software	After mapping paired-end reads with Bowtie2 (v 2.4.2), were then merged and sorted with Samtools, and PCR duplicates were removed using the Picard MarkDuplicates tool. Subsequently, SAM files were converted to BAM files, indexed, and processed to generate bedGraph and bigWig files using bamCompared from DeepTools2. The resulting bigWig files were visualized using Integrative Genome Viewer (IGV), and bedGraph files were visualized using MATLAB (2015) and used for correlations. Peak calling was performed with MACS2 callpeak

Flow Cytometry

Plots

Confirm that:

- [x] The axis labels state the marker and fluorochrome used (e.g. CD4-FITC).
- [x] The axis scales are clearly visible. Include numbers along axes only for bottom left plot of group (a 'group' is an analysis of identical markers).
- [ ] All plots are contour plots with outliers or pseudocolor plots.
- [x] A numerical value for number of cells or percentage (with statistics) is provided.

Methodology

Sample preparation	For ChIP-seq, cells were grown until ESP, and samples crosslinked with 1% Formaldehyde, quenched with Glycine 250mM, washed and resuspended in PBS 1X.<br>For Hi-C, cells were grown until ESP, and samples crosslinked with 1% Formaldehyde, quenched with Glycine 250mM, washed and resuspended in PBS 1X<br>For RNA-seq, cells were grown until ESP, and samples fixed with ice cold 4% Formaldehyde, washed and resuspended in PBS 1X + 0.01 U/µl SUPERase-In RNAse inhibitor.<br>To quantify the percentage of GFP+ cells, cells were fixed as for Hi-C
Instrument	For cell sorting: MoFlo AstriosEQ cell sorter (Beckman-coulter)<br>For analysis of GFP+ cells: CytoFLEX S (Beckman-coulter)
Software	Kaluza analysis software.
Cell population abundance	After cell sorting samples expressing GFP+ were enriched to approximatelly 80% and GFP- samples were enriched at >95%, (Supp Fig 1). This was determined using MoFlo AstriosEQ cell sorter (Beckman-coulter).
Gating strategy	A first gate was made on a Forward Scatter (FSC-Height) - Side Scatter (SSC-Height) dot plot to select the bacterial population. Doublets were discarded using an SSC-Area – SSC-Height dot plot. Finally, the cut-off of GFP positive cells was determined by comparing the PprgH-gfp sample to a control bacteria sample, using a 525/52-nm bandpass filter for collecting fluorescence emission signal.

- [ ] Tick this box to confirm that a figure exemplifying the gating strategy is provided in the Supplementary Information.
